# Motivation dynamically increases noise resistance by internal feedback during movement

**DOI:** 10.1016/j.neuropsychologia.2018.07.011

**Published:** 2019-02-04

**Authors:** Sanjay G. Manohar, Kinan Muhammed, Sean J. Fallon, Masud Husain

**Affiliations:** a Nuffield Department of Clinical Neurosciences, John Radcliffe Hospital, University of Oxford, Level 6 West Wing, OX3 9DU, United Kingdom; b Department of Experimental Psychology, 15 Parks Road, Oxford, United Kingdom

**Keywords:** Motivation, Saccades, Noise, Reward, Oculomotor, Optimal control theory, Eye movements, Cognitive effort

## Abstract

Motivation improves performance, pushing us beyond our normal limits. One general explanation for this is that the effects of neural noise can be reduced, at a cost. If this were possible, reward would promote investment in resisting noise. But how could the effects of noise be attenuated, and why should this be costly? Negative feedback may be employed to compensate for disturbances in a neural representation. Such feedback would increase the robustness of neural representations to internal signal fluctuations, producing a stable attractor. We propose that encoding this negative feedback in neural signals would incur additional costs proportional to the strength of the feedback signal. We use eye movements to test the hypothesis that motivation by reward improves precision by increasing the strength of internal negative feedback. We find that reward simultaneously increases the amplitude, velocity and endpoint precision of saccades, indicating true improvement in oculomotor performance. Analysis of trajectories demonstrates that variation in the eye position during the course of saccades is predictive of the variation of endpoints, but this relation is reduced by reward. This indicates that motivation permits more aggressive correction of errors during the saccade, so that they no longer affect the endpoint. We suggest that such increases in internal negative feedback allow attractor stability, albeit at a cost, and therefore may explain how motivation improves cognitive as well as motor precision.

## Highlights

• Motivation can increase speed and reduce behavioural variability.
• This requires stabilising neural representations so they are robust to noise.
• Stable representations or attractors in neural systems may come at the cost of stronger negative feedback.
• Examination of trajectory correlations demonstrates that reward increases negative feedback.
• We propose that the cost of stabilising signals explain why effort is expensive.

## 1 Introduction

Motivation allows us to be both fast and accurate at the same time, violating the speed-accuracy trade-off. For example, if we stand to win or lose, we can act both quicker and more accurately. In motor control, the prospect of reward not only increases the speed of movement, but also reduces motor endpoint error (Takikawa et al., 2002, Manohar et al., 2015). Similarly, in tasks that require cognitive control, motivation can shorten reaction time but simultaneously reduce errors (Boehler et al., 2012, Chiew and Braver, 2013). Such findings contravene traditional optimal control theory, which predicts that speed should trade off with accuracy, because moving faster requires larger motor control signals (Harris and Wolpert, 1998, Todorov, 2004). Larger signals not only incur greater noise and are therefore more variable, but also have higher energetic costs. These costs of moving fast can be offset by the time saved, so that there is an optimal speed of movement (Berret and Jean, 2016, Choi et al., 2014). But then how could motivation improve accuracy despite faster movement?

We proposed that the effects of neural noise could be reduced, but at some cost (Manohar et al., 2015). According to this view, noise limits performance (Faisal et al., 2008), but when reward is available, it is economical to invest in precision by attenuating the effects of noise. Motivation thus modulates our level of performance because suppressing effects of noise is itself costly, and this cost explains why precise motor or cognitive control is only exerted when required. The optimal investment in compensating for noise can be described by a cost function that includes not only a time penalty and an error penalty, but also the cost of noise-compensatory processes. If reducing the effects of noise were costly, then optimal behaviour will be to improve precision when available reward is higher. But this leaves an important question open: how might the effects of neural noise be attenuated?

**Fig. 1:**
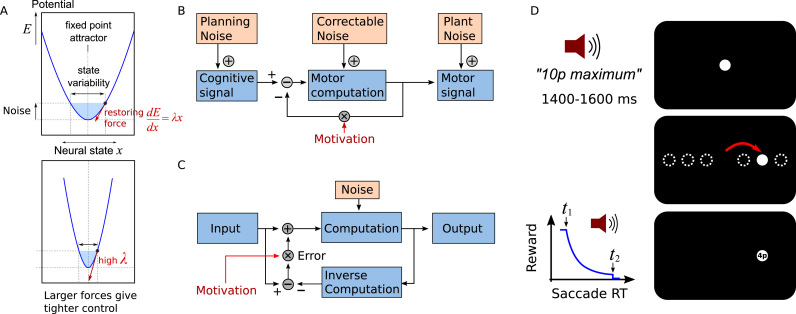
**Can the effects of internal noise can be attenuated by motivation?** A) Neural patterns representing information can be held stable by negative feedback (red arrows), producing an attractor state, represented as a potential energy well (solid blue line). Even in a stable state, representations are perturbed by noise, producing natural variability in the neural state (shaded blue area). The slope of the sides determines how aggressively noise is corrected. By increasing the gain of the negative feedback *λ*, variability can be reduced. The internal neural feedback is analogous to a restoring force in motor control. B) Model of oculomotor system. When an action is produced, a cognitive signal gives rise to a motor command, through some computation (e.g. in the case of eye movements, a combination of temporal filters, Coubard, 2013). Noise can potentially enter the system at three points: 1) at cognitive or planning stage, 2) in the course of the computation within the negative feedback loop, or 3) after the output has been computed (Eggert et al., 2016). Noise entering within the closed loop will manifest in downstream processes such as motor execution, but resulting error can be subsequently corrected during the computation. Due to the structure of this loop, feedback can only compensate for noise arising at the middle stage. We suggest that motivation (e.g. by reward) will allow the strength of feedback to be increased, ultimately reducing motor variability. Symbols “+” indicate additive combination of signals, “−” indicates a comparator presumably implemented via inhibitory interneurons. C) The oculomotor controller is an instance of a more general feedback controller: it is effectively a scalar linear system, so errors can be corrected by direct subtraction. More complex computations can also be corrected by negative feedback, though error signals must be generated by a backward computation. Motivation could amplify the compensation for noise, to reduce variability in the output signal. D) Participants performed an incentivised prosaccade task. After fixating the centre of the screen, participants heard a voice indicating the amount of monetary reward on offer for the upcoming movement. After 1400–1600 ms the light moved to one of 6 target locations, indicating that a saccade had to be made towards that location. When gaze reached the target, reward was calculated using an adaptively adjusted exponential falloff, to keep overall reward rate approximately constant. The measures of interest are saccade velocity, endpoint variability, and variation in the trajectory. (For interpretation of the references to colour in this figure legend, the reader is referred to the web version of this article.)

One possible mechanism is to hold information in a more stable, robust state. Any physical system that returns to a stable state after a small perturbation is described mathematically as having an “attractor” (Milnor, 1985). Systems with attractors tend to settle or gravitate into a subset of the possible states, depending on the initial state of the system. For an attractor to be stable, small disturbances – for example due to noise – must be cancelled out by pushing the system towards its stable state (Fig. 1**A**). In the case of a ball in a well, for example, gravity and friction act on the surface to produce a reactive force that opposes the perturbation, which is mathematically equivalent to negative feedback. Similarly, some patterns of neuronal activity may also be stable, such that if the pattern is disturbed by external input or by internal noise, it will tend to return to those stable attractor patterns (Sebastian Seung, 1998). The relative stability of a system's states can be described mathematically by a potential energy for each possible state, and an attractor corresponds to a ‘well’ or local minimum in this potential. The steepness of the walls, corresponding to the energy gradient, indicates how strongly the system is pushed back to the stable state.

Stability operates at a number of levels in the brain. Isolated neurons typically have a stable attractor at their resting membrane potential, and deflections from this state are corrected by negative feedback currents (FitzHugh, 1961), which ultimately dissipate the electrochemical gradient and are thus energy-expensive. In larger systems of neurons, the statistics of neural activity can form stable active states, such as persistent (Wilson and Cowan 1972), rhythmic (Wang, 2010) or chaotic neural firing (Miller, 2016). Such stable states have been proposed to maintain task-relevant information in a wide range of cognitive domains, including working memory (Almeida et al., 2015), decision-making (Mante et al., 2013, Sussillo, 2014, Wang, 2002), navigation (Samsonovich and McNaughton 1997) and task rules (Armbruster et al., 2012). In each of these cases, it is proposed that computations involve cognitive signals that carry information about decision variables, goals, or state representations, which are all susceptible to neural noise. Negative feedback from inhibitory interneurons corrects for perturbations by pulling the system back to a stable state.

Could stabilising such cognitive signals against noise be costly? Top down signals can reduce variance in the activity of a populations of neurons, which can be modelled by increasing the gain of inhibitory interneurons (Kanashiro et al., 2017). In this particular case, greater stability comes at the cost of increasing the amplitude of feedback signals. For continuous attractor networks, increasing the peak firing rate reduces noise accumulation (Burak and Fiete, 2012). Although there are several ways to increase network stability, such as increasing the steepness of tuning curves or the nonlinear transfer function, simulations again indicate that attractors can be stabilised at the cost of greater neural activity. More generally in any physical system, there are entropic costs for stabilising internal states using negative feedback (Lan et al., 2012), because immunizing against noise requires information to be removed from the system, consequently dissipating energy (Sobczyk and Hołobut, 2012, Touchette and Lloyd, 2004).

Noise compensation based on internal feedback could be considered a type of “reactive” cognitive control, whereas setting a higher gain for this feedback in advance corresponds to “proactive” control (Braver, 2012). Both types of control may be subjectively effortful and objectively costly, but there is no consensus as to why (Botvinick and Braver, 2015). Presumably, more difficult computations are more costly in terms of interference, time and metabolism (Kurzban et al., 2013, Shenhav et al., 2017). Our behaviour may depend on learned estimates of the costs (Lieder et al., 2018), but any fundamental metabolic costs of precision remain difficult to quantify. In this paper we develop an attractor-precision model of effort, and offer a preliminary test of the theory in the domain of motor control, using oculomotor control as a model system.

Eye movements offer an ideal opportunity to study how motivation affects behavioural variability, because they provide a precise moment-by-moment readout of internal processes. Saccades are generated by well-studied brainstem circuits (Munoz et al., 2000, Sparks, 2002), yet cognitive signals such as reward can improve both their speed and accuracy (Takikawa et al., 2002, Muhammed et al., 2016).

Standard models of oculomotor control describe how a visual signal from the superior colliculus is converted into a motor command consisting of a phasic duration-modulated burst to accelerate the eye, and a subsequent tonic component to maintain the new eye position (Optican, 2005, Sparks, 2002). These models use a negative feedback loop to keep track of discrepancies between the actual motor output and the desired output, to prevent accumulating noise (Fig. 1**B**) (Robinson, 1981). Saccades are so fast that the rapid online trajectory adjustments cannot be controlled by external sensory feedback (Mays and Sparks, 1980, Quaia et al., 2000), but rather must utilise internal feedback from the outgoing motor signals (Chen-Harris et al., 2008, Joiner et al., 2010, McSorley et al., 2004). Thus online noise correction for saccades operates primarily at the level of internal representations, and so could potentially serve as a model for cognitive control.

Online trajectory correction allows us to distinguish the effects of neural noise entering at different stages of eye movement control (Eggert et al., 2016). Some trajectory variability is due to planning or target location uncertainty (planning noise), whereas other variation arises from in-flight motor noise (plant noise) (Beers, 2007). Noise occurring within the feedback loop can be corrected during the course of the movement. This means that the across-trial correlation of eye position over time can reveal in-flight noise-correction – a method previously used for limb trajectories (Gordon and Ghez, 1987, Messier and Kalaska, 1999). In the present study, we use this principle to test whether motivational improvements by reward are mediated by the rapid internal correction of noise.

First we simulate correction of noise by linear negative feedback, showing its characteristic effect on correlations over the trajectory of internal states. Second, we examined how motivation alters saccade trajectories. We predicted that motivation would energise movements and reduce variability as previously observed, but would also show a signature of stronger negative feedback, manifest in the correlation over the trajectory. Finally, we fit an established model of noise sources to the saccades, making the prediction that motivation would increase the gain of negative feedback in the saccade generator.

## 2 Methods

### 2.1 Feedback control model

In order to understand how motivation could improve the precision of representations, we used optimal control theory, which is often formulated in the domain of motor performance. Optimal control theory provides an elegant framework to determine what motor control commands are needed to keep a system close to a desired state, in the face of noise (Supplementary material). The control command signals are treated as energetically expensive, which leads to a trade-off between the benefits of rapidly cancelling perturbations, and the costs of sending large control signals. We treat the cost as proportional to the squared control signals, in analogy with the physical effort cost which is approximately related to energy consumption. Since these corrective signals are energetically costly, they must also be balanced against potential rewards, the cost of time, and the cost of errors, to achieve an optimum balance between speed and error (Choi et al., 2014, Harris and Wolpert, 2006, Shadmehr et al., 2010b). Crucially, when rewards are higher, it becomes optimal to invest in stronger feedback control (Fig. S11B). By estimating the relative value of reward and cost of noise-compensating feedback signals, the motivational improvement can be derived (Fig. S11C).

The very same principles can be applied to the control of internal *neural* states. If a computation – for example the transformation of a signal – is perturbed by noise, deviations in the output can be detected and corrected (Quaia et al., 2000) (Fig. 1**B**). However, without sensory feedback, the system can only ‘notice’ and subtract noise if it can compare its output with its input: either it must obey simple linear dynamics, or else it must invert the transformations it is trying to compute (Fig. 1**C**) (Shadmehr et al., 2010a). We suggest that correction for internal noise in cognitive processing would also incur a cost, e.g. proportional to the squared correction command signals.

Numerical simulations were run to confirm the predicted effects of noise on autocovariance of states over time, i.e. the degree to which variance at one moment in time explains variance at another moment. We used a very simple first-order linear controller, which tries to keep a single scalar variable fixed (Supplementary material), with the form *dx* = (ε – *λx*) *dt*. The system's state *x* accumulates noise ε, which can be corrected by feedback with gain *λ*. In order to examine the difference between simply reducing the level of noise, versus increasing the gain of online negative feedback, four conditions were simulated: 1) noise without feedback, 2) noise with feedback, 3) reduced noise, and 4) noise with increased feedback. In each condition, the time-time covariance and correlation of *x* across 1000 simulated trials was computed.

### 2.2 Effect of motivation on saccade trajectories

#### 2.2.1 Participants

21 healthy volunteers were recruited, age range 22–32 years (mean 27). All gave informed consent according to the Declaration of Helsinki and the study was approved by the Oxford local research ethics committee. Participants were instructed that they had to make rapid eye movements to the lights that came on, in order to obtain monetary rewards. They were told that the quicker they looked at the target, the more of the available reward they would win, on each movement. They were instructed they would be paid based on their performance (see below for calculation).

### 2.3 Materials

Participants were seated at a tower-mounted Eyelink 1000 sampling monocularly at 1 kHz, stabilised with both chin-rest and forehead-rest 60 cm from the screen. Nine-point calibration was performed at the start of the experiment, and fixation position was zeroed at the start of each trial. Stimuli were displayed on a 17-in. CRT display with 1024 × 768 pixels scanning at 100 Hz controlled by a PC running Matlab and PsychToolbox.

### 2.4 Task

At the start of each trial, a white disc at the centre of the screen, 1 degree radius, shown (Fig. 1**D**). Participants were required to fixate the illuminated disc until gaze remained stable within the target with no saccades for 500 ms. Then, one of three possible spoken auditory reward cues was played via speakers, indicating the amount of reward that was available on this trial. Cues were “zero pence maximum”, “ten pence maximum”, and “fifty pence maximum”, with matched peak sound amplitude, lasting 1200 ms. After a variable foreperiod between 1400 and 1600 ms, a target disc (also white, 1 degree radius) was shown at one of six possible target locations, and simultaneously the fixation disc was extinguished. The possible targets were to the left and right at 4, 8 and 12 degrees either side of the screen centre, in a horizontal line (Fig. 1**D**). The target remained illuminated until gaze arrived within 3 degrees of the centre of the target disc. As soon as the eye position was detected to be at the target, the response time was recorded relative to target onset, and was used to calculate reward. Reward was determined by an exponential falloff with response time, scaled by the maximum reward possible on that trial (0p, 10p or 50p). Response time was defined as the time from the target onset, until the eye came to rest at the target, and so comprises a reaction time, of the order of 185–330 ms (95% confidence interval) and a briefer movement time (27–70 ms). This composite measure was used because of difficulty estimating the true reaction time (time until start of saccade) online. When measuring the effects of incentives, it is important to keep reward rate relatively constant across participants and through the course of the experiment, because reward rate can influence oculomotor performance (Manohar et al., 2017). Therefore an adaptive procedure was used to determine the reward. The start-time and falloff-time of the reward function were updated on each trial based on the response times on the previous 20 trials, so that 10% of trials were rewarded at the maximum, and 10% of trials were rewarded at zero, and consequently 25% of the maximum was obtained on average.

$$R=R_{\max}*\exp\left(-\left(\mathrm{RT}-t_{0}\right)/\left(t_{1}-t_{0}\right)\right)$$

where *t*_0_ and *t*_1_ are the 0.1 and 0.9 quantiles of the RTs on the most recent 20 trials. Numerical reward was displayed at the target location. Also, if the reward exceeded 10p, a “ping” sound was played, and if it exceed 40p, a cash register “ker-ching” sound was played.

The experiment began with 5 practice trials. This was followed by 15 blocks of 54 trials each, separated by brief breaks. All 3 target distances and 3 reward levels were randomly intermixed. Thus each participant performed 810 trials, with 90 trials for each amplitude and reward level. The experiment lasted approximately 55 min.

### 2.5 Analysis

Saccades were grouped by target distance, and eye position was projected onto the horizontal axis. Saccades were parsed using standard criteria (velocity > 30deg/s, acceleration > 9500deg/s^2^) to find the first eye movement that was larger than 1 degree, for each trial. For each subject and amplitude condition, trials were discarded if the eye position deviated on average by 1.2 degrees from the mean eye position trace. Trials were also excluded if the saccade duration was more than 2 sd. below the mean, if there was loss of eye tracking during the saccade, or if the jerk (d^3^*x*/d*t*^3^ calculated in 4 ms windows) exceeded 0.045deg/s^3^ at any point during the saccade (indicating an artefact or partial blink). Peak saccade velocity was calculated by estimating the gradient in 3 ms windows, and taking the maximum during the saccade. Saccade endpoint variability was calculated as the standard deviation of the horizontal eye position at the end of the saccade (Beers, 2007), calculated for each reward and target distance condition. Raw data for the 12-degree condition for all 21 subjects is presented in Fig. S1.

Saccade velocities and amplitudes were first examined using a 2-way repeated-measures ANOVA, with three levels of reward and three target distance conditions.

The relation between saccade amplitude and velocity (i.e. the ‘main sequence’) was estimated for each subject and target distance condition, by regressing the saccades’ peak saccade velocities against their amplitudes with a linear model (method illustrated in Fig. S4). To ask whether reward altered this relationship, the mean of the residuals of this model were calculated for each reward condition. If the residuals were positive for a reward condition, this indicated that saccades of a given amplitude had a higher velocity in that reward condition, relative to other amplitude-matched saccades in other reward conditions. The residuals were therefore an index of how reward increased velocity after correcting for amplitude effects.

To plot the main sequence, for each subject and target distance condition, trials were binned according to their amplitude using a sliding window of 20% of trials. For example, for subject 1, saccades in the 20–40 percentile bin (i.e the second-smallest one-fifth of that subject's saccades) were grouped to get a mean amplitude and mean velocity. The corresponding bins for each subject (i.e. their own 20–40 percentile amplitude bin) were plotted as a single point, using the across-subject mean peak velocity and standard error, at an x-axis value calculated as the mean amplitude across subjects for that percentile bin. This corrects for the fact that individuals have different preferred amplitudes and velocities, without making assumptions about the shape of the distribution.

For subsequent analysis of the mean and covariance of trajectories, saccades were stretched or compressed to a uniform duration. This was achieved by resampling each saccade to 50 timepoints by linear interpolation.

### 2.6 Fitting of noise components

To complement the model-free examination of variability, we fitted the model of Eggert et al. (2016) to the eye movement traces, which estimates the contribution of three noise sources and the negative feedback gain parameters given the mean, variance and covariance of eye position trajectories (Fig. 5**A**).

This model utilises the mean eye position as a function of time to estimate control signals, assuming standard time constants of the oculomotor plant (i.e. the mechanical eye-and-muscle system). These signals are then used to estimate the across-trial variability of eye position that would be expected if noise were injected at various points in the system. To do this, we used analytical equations for the predicted variance and endpoint covariance as a function of time (Supplementary material), which depend on four free parameters: the quantity of planning/input noise, premotor burst neuron noise, oculomotor neuron/output noise, and the feedback gain. The model's predicted variance and covariance at each time bin, for a given set of noise parameters, was approximated using numerical convolution and integration, utilising the mean position trace for each condition. The predicted values can then be compared with the empirically observed variance and covariance (Eggert et al., 2016). For each reward condition, we fitted these four parameters to minimise the discrepancy between the empirical and predicted variance and covariance. We examined the farthest target distance condition, which most closely matched the data from Eggert et al. (our mean amplitude was 11.8, compared to 10.1deg in their study). Our time parameters were τ_1_ = 223 ms, τ_2_ = 14 ms, τ_3_ = 8 ms, delay = 4 ms. Note that we used a τ_3_ that was 4 ms longer than Eggert et al., because this was observed to give considerably better fits to the trajectories, as measured by squared error, and as judged by the timing of the peak variance. For each of the four parameters, we tested the linear effect of reward on the parameter value using a repeated-measures model (random intercept per subject, fixed effect of reward) yielding a t-statistic for each parameter. Before statistical analysis, the gain parameter was log-transformed as it was non-normally distributed.

Note that this analysis is not independent of the conclusions above. Rather, it verifies the intuition provided by the simulations, that in order to reduce the end-saccadic covariance with earlier timepoints despite increased mid-saccadic variability, a higher feedback gain is required. The model-based approach demonstrates this intuition holds even when the plant has more complex dynamics, and pre-feedback and post-feedback noise sources are included.

## 3 Results

### 3.1 Simulated autocorrelations with negative feedback control

**Fig. 2:**
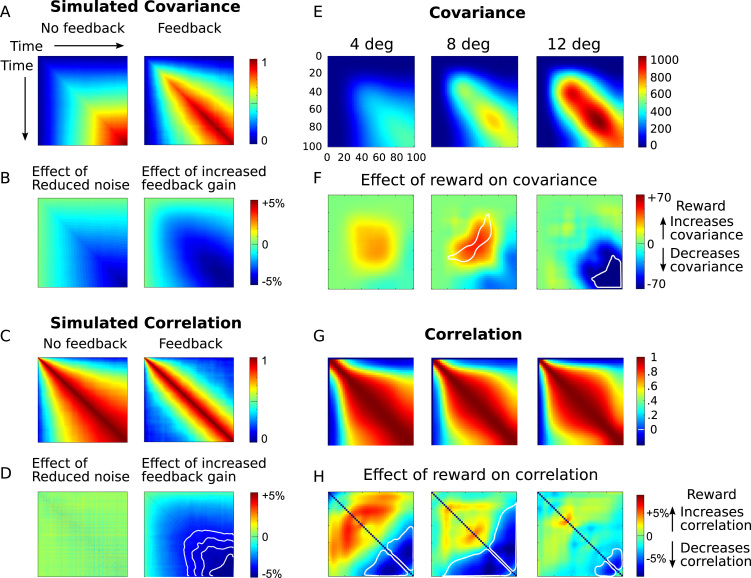
**Simulated and observed patterns of covariance and correlation**. Panels A–D show simulations of a linear system with negative feedback. Panels E–F show data from eye movement trajectories. A) Left: The time-time heatmap shows the covariance in the state of the simulated system between each pair of timepoints. Colours indicate the degree to which the state at each moment in time explains the state at each other moment in time. A system that simply accumulates uncorrected noise demonstrates increasing variance. Right: Introduction of a negative feedback causes distinctive changes in the covariance and correlation structure, such that the state at late timepoints is less correlated with previous timepoints. B) Left: Reducing noise causes reductions in covariance, that scale linearly over time. (Colour indicates covariance difference for low noise minus high noise simulations.) Right: However strengthening the negative feedback yields a different pattern, with the strongest reductions between later timepoints. (Colour indicates difference between low feedback and high feedback gain.). C) Left: In a simple linear system, correlation coefficients between pairs of timepoints increases over time, as noise accumulates (Colour indicates Pearson correlation coefficient). Right: introducing negative feedback reduces correlation between states separated in time. This is because noise in previous states is cancelled out. D) Left: Reducing noise does not affect the correlation, since it simply scales down the total variance at all times. (Effect of high-feedback minus low-feedback shown, as the difference in z-transformed correlation coefficients.) Right: Strengthening the negative feedback reduces correlations between later pairs of timepoints. Lines indicate isopleths of reduced correlation. E) The covariance plot shows how well saccade position at each moment in time explains variance at each other moment in time. Warm colours indicate a positive covariance, i.e. on trials where the eye position is closer than average to the target at time *t*_1_, it is also closer than average at time *t*_2_. Eye position is most variable (across trials) at the middle or end of the saccade. The diagonal indicates the variance at a moment in time. Since moments close together in time will have strongly correlated eye positions, areas near the diagonal are warm. Online error correction is indicated by a low covariance between early and late timepoints, despite significant variance early in the saccade. F) Cool colours indicate times when reward reduces covariance. Reward reduces both variance (diagonal) and covariance (off-diagonal), at the end of a saccade. This could be due either to lower time-time correlation, or due to lower absolute variability, with reward. G) Time-time correlation plot factors out the overall variability, concentrating on how well variations at one moment in time predict variations at another moment. Cooler colours near the lower and right edges indicate that early variations do not predict later variations, characteristic of negative feedback. H) Reward significantly reduces correlations between the final eye positions, and preceding eye positions (dark blue areas). This can be interpreted as increased negative feedback towards the end of the movement. Contours indicate significant effects of reward on the correlation, after correction for multiple comparisons. (For interpretation of the references to colour in this figure legend, the reader is referred to the web version of this article.)

We simulated negative feedback control to maintain a scalar variable at a point attractor, subject to noise. As noise accumulates, the variance increases, and autocorrelation between neighbouring time-points is observed (Fig. 2**A&C**, left). The simulations demonstrated that introducing negative feedback results in a qualitative change in the covariance and correlation pattern (Fig. 2**A&C**, right). As the strength of feedback is increased, there is a hallmark reduction in autocorrelation with earlier timepoints, that is strongest later in time (Fig. 2**B&D**, right). On the other hand, simply reducing the level of input noise reduced the covariance but had no effect on the correlation (Fig. 2**B&D**, left). Therefore, correlation patterns differ when there is online noise correction, compared to when the input noise is simply reduced.

Next we tested whether empirical trajectories of saccadic eye movements matched these simulations.

### 3.2 Incentives increases saccade amplitude and velocity

**Fig. 3:**
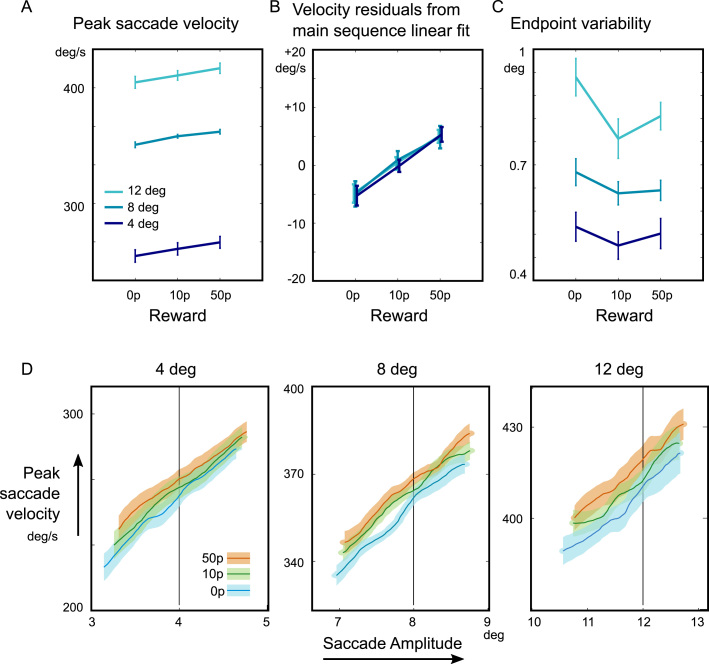
**Reward increases velocity and endpoint precision of saccades**. A) The mean peak saccade velocity of saccades increased with target distance as expected. Velocity also increased with larger incentives. B) To investigate whether reward effects on velocity could be explained by concomitant increases in the saccade amplitude, the velocity residuals after regressing out the individual saccade amplitudes, for each target distance condition, were grouped by reward (Method in Fig. S4). The mean residuals indicate deviation from the main sequence. Velocity was increased independently of amplitude. C) Saccade endpoint variability was calculated as the standard deviation of the horizontal eye position. The mean variability was greatest for the farthest targets, but decreased with reward, indicating more precise oculomotor control. D) The main sequence is shown for the nine conditions. For each participant, reward and target distance, saccades were binned by the quantiles of the saccade's amplitude, and the mean peak velocity in each 20% bin was calculated. This method ensures that each bin represents the same number of saccades, equally drawn from each subject. The x-coordinates represent the mean amplitude for that quantile bin, across subjects. The graph shows the mean across subjects of this binned velocity as a function of amplitude. Error bars and shaded error areas indicate the within-subject standard error of the mean.

Mean peak saccade velocity increased with reward (Fig. 3**A,** Table S1, 3 × 3 repeated measures ANOVA, main effect of reward F(2,160) = 5.26, p = 0.0061, no interaction with target distance). However, saccade amplitudes also weakly increased with reward (Table S1, average increase 0.10deg ± sem 0.04, F(2,160) = 3.14, p < 0.046, no interaction with target distance). It is well known that saccade velocity increases in direct proportion to the amplitude of the movement, a relation known as the ‘main sequence’ (Bahill et al., 1975). To investigate whether this amplitude increase could account for the faster movements, we tested whether the main sequence differed as a function of reward. A linear regression of peak saccade velocity against amplitude was performed, and the residuals from this regression were obtained (Fig. 3**B**). The residuals differed significantly by reward level (F(2,160) = 52.9, p < 0.001). Residuals were significantly positive on high-reward trials for all 3 amplitudes (t(20) = 3.49, 3.27, 4.93, p = 0.002, = 0.004, < 0.001 respectively), and negative for the low-reward trials (t(20) = 3.26, 2.79, 2.71, p = 0.004, 0.011, 0.013 respectively), indicating that reward increases velocity over and above that predicted by the main sequence. Thus increasing reward weakly increased amplitude, but increased velocity over and above what would be predicted from this amplitude increase alone. This effect was visualised by plotting saccades’ velocity against amplitude (Fig. 3**D**). To compare across subject, we used a sliding window according to amplitude quantiles. The mean velocity in each amplitude window (width 20% quantile) is calculated, and the mean across subjects is plotted, with the standard error of the amplitude effect. Although reward increased both amplitude and velocity, it had no net effect on saccade duration (3 × 3 mixed model, no main effect of reward on duration F(1,185) = 0.22, p > 0.05; no interaction with target distance F(1,185) = 0.18, p > 0.05).

### 3.3 Incentives reduce endpoint variability

The standard deviation of the endpoint position was examined for each subject, reward and distance condition (Fig. 3**C**). This quantifies the error in saccade planning, relative to the average saccade endpoint. We find that reward weakly decreased saccade endpoint variability (F(2,160) = 3.38, p = 0.037), despite increasing the overall amplitude. Post-hoc pairwise comparisons demonstrated that the difference was significant between the lower two reward levels (main effect of 0p vs 10p F(1,122) = 6.34, p = 0.013) but not between the higher pair (10p vs 50p, F(1,122) = 0.8, p > 0.05). Saccade endpoint variability usually increases linearly with the amplitude of saccades (Abrams et al., 1989). If we scaled variability to be relative to saccade amplitude, this further strengthens the findings, because reward also increases saccade amplitudes, and so the variability per unit of amplitude will be even smaller. Moreover, saccades with faster peak velocities usually also have greater endpoint variability (Harris and Wolpert, 1998). This complements the empirical finding that movements to smaller targets are slower (Fitts’ law, Fitts, 1966). In contrast to what one might expect from this law, motivation reduces variability despite an increase in speed. Next, we asked whether motivation's effects on improving performance result from reduction of the effects of internal noise, by examining saccade trajectories.

### 3.4 Saccade trajectories are modulated by reward

**Fig. 4:**
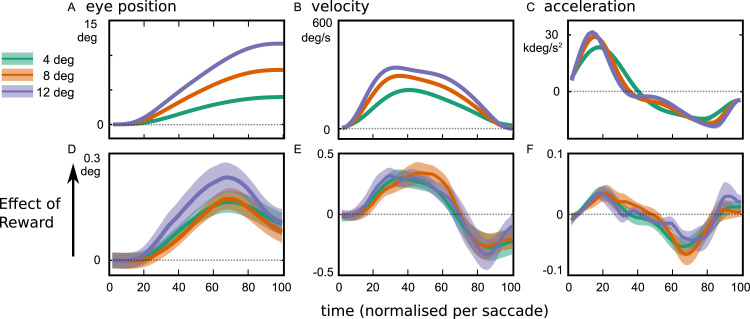
**Saccade trajectories as a function of target distance, and the effects of reward**. The top row shows the grand average trace of saccade position, velocity and acceleration across all participants and reward levels, split by target distance. Saccades were normalised to 100 time-units for averaging, with the average duration being 30–60 ms. Positive values are in the direction towards the target. When the target is further away, saccades A) are greater in amplitude, B) have faster peak velocity and C) have a larger peak acceleration in the first 10 ms. Reward D) advances saccades trajectories towards the target, E) increases peak velocity early in the saccade, but slows the eye down earlier, and F) boosts the initial acceleration and final deceleration.

We plot the horizontal eye position, velocity and acceleration as a function of normalised time, for each amplitude condition (Fig. 4**A–C**). These traces are calculated by stretching the saccades to all last 100 time units (see Methods). The average saccade duration was 34 ± 4 ms, 48 ± 4 and 58 ± 5 ms (mean ± s.d.) for the three target distances. To show the effect of reward, we take the high-reward condition minus low-reward condition, for each of these traces (Fig. 4**D–F**). Reward pushed saccades towards the target, most strongly at the midpoint of the saccade (Fig. 4**D**). Faster movements were manifest by an increase in velocity early in the saccade, and a reduction in velocity at the end of the saccade (Fig. 4**E**). The increased impulse was indicated by greater acceleration at the start of the saccade, and greater deceleration at the end of the saccade (Fig. 4**F**).

Next we enquired how motivation reduces endpoint variability. According to traditional models of saccade generation, there are three kinds of noise that can be distinguished (Fig. 1**B**) (Eggert et al., 2016). First, *planning noise* causes each saccade to be planned as though it had a different desired endpoint, from the outset. Second, *plant noise*, e.g. in the nerve or muscle itself, results in variability that builds up throughout the movement. Finally, noise injected within the feedback control loop causes a transient deviation of the saccade from its planned course, but this error can be corrected in-flight by immediate feedback control loops. This kind of *correctable* noise should result in variability across trials that does not correlate with the actual endpoint – indicating that motor error is subsequently corrected.

To quantify variability, we examined the across-trial variance in instantaneous eye position, as a function of time during the saccade, and the corresponding covariance between the instantaneous eye position at each pair of timepoints (Fig. 2**E**). The effect of reward on this matrix was examined using a linear effect of reward across subjects, with the model cov_*tt*_ ~ reward + (1|subject), for each target distance separately, indicating that we permitted a random intercept for each subject, in addition to a linear influence of the three reward levels. This gave a matrix of the reward effect on covariance for each pair of times (Fig. 2**F**), matching the structure of the covariance plot, together with a *t*-statistic. Reward weakly reduced the endpoint variance and covariance towards the very end of saccades, in the farthest target condition. White lines indicate regions where the reward coefficient of the linear model was significant at p < 0.05, and a smaller region was significant at p < 0.0004 (stringent threshold determined by permutation test controlling for false-detection rate across the three matrices). Note that reward could reduce the covariance either by reducing the total variance, or by reducing noise autocorrelations over time.

To factor out absolute changes in variance, and focus only on correlations – i.e. the degree to which earlier eye position predicted later eye position, we examined the time-time correlation matrix of eye position (Fig. 2**G**). In this plot, warmer colours indicate that eye position at one moment in time positively predicts eye position at another moment in time, across trials. The difference between these plots show the effect of reward on the correlation (Fig. 2**H**, calculated using linear model as above, but using the Fisher inverse hyperbolic tangent z-transformed Pearson moments). Significant reductions in correlation are shown with a white outline, and in the 4- and 8- degree conditions, smaller regions remained significant after stringent correction for false detection rate. This indicates that reward reduces the noise correlation between earlier timepoints during the saccade, and late timepoints. This pattern is a hallmark of in-flight negative feedback control over trial-to-trial variability in the trajectory, and matches the simulations of increased feedback gain (Fig. 2**D**). The size of this reward effect predicted, across subjects, the effect of reward on endpoint error, suggesting that the negative feedback is indeed instrumental in reducing endpoint variability (Fig. S5). The finding is therefore consistent with the view that motivation attenuates the effects of noise online during the saccade.

We also looked for effects of motivation on the fixation period before the saccade. No significant effects were found on microsaccades, the power spectrum of ocular movements, or on ocular drift (Fig. S2).

### 3.5 Fitting of noise components

**Fig. 5:**
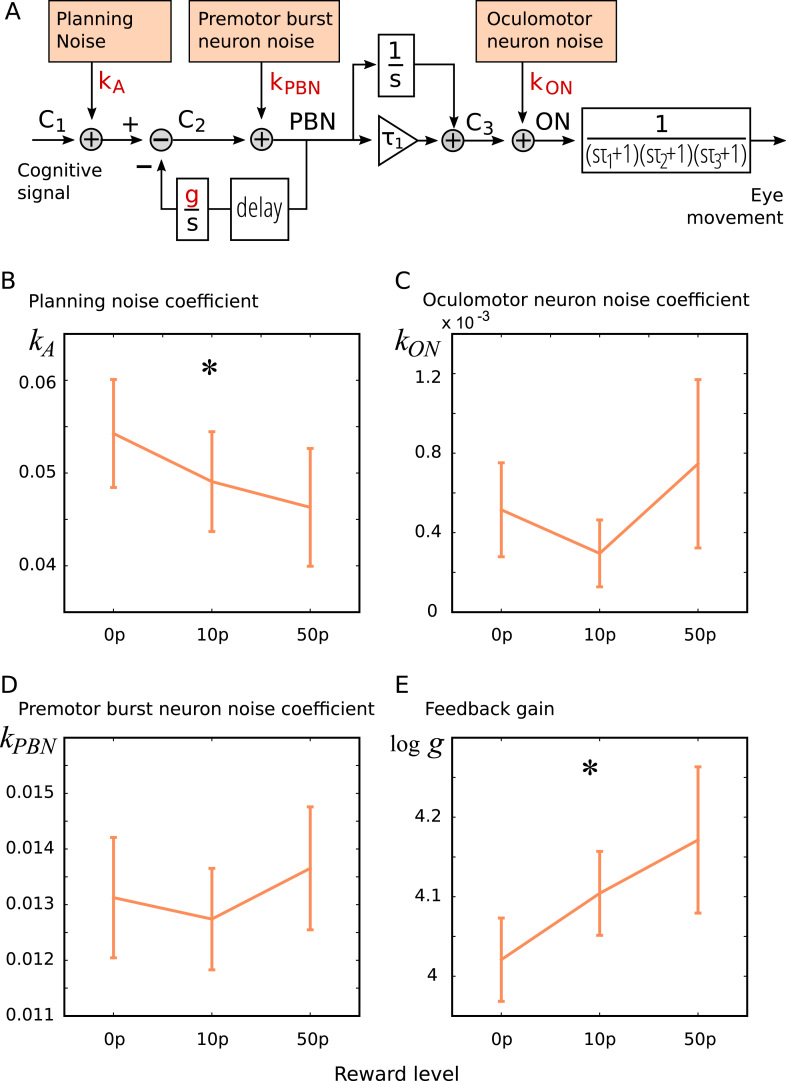
**Fitting noise components to saccade trajectory variability**. A) We fitted a model of saccade variability to the data. The model assumes a saccade generator with a negative feedback loop, and three noise sources: variability in the input signal, noise within the feedback loop, and noise after the feedback e.g. in the oculomotor neurons and eye muscles (plant). The control signals C1 C2 and C3 are estimated from the mean saccade trajectories, and the plant time constants τ_1_ τ_2_ and τ_3_ are taken from Harris and Wolpert (1998). It has four fitted parameters, which we estimated for each reward condition, for the large amplitude condition which matches previous models). The parameters are noise coefficients for planning noise, premotor burst neuron noise, and oculomotor neuron noise, plus the gain of negative feedback. B–E) Planning noise was significantly reduced by reward. There was no effect of reward on oculomotor neuron noise (which includes plant noise) or premotor burst neuron noise, but reward increases the gain of the feedback (y axis shows log of gain).

The analytic model (Fig. 5**A**) yields four parameter estimates per participant and per reward level: the coefficient of planning noise, premotor burst neuron noise, oculomotor neuron noise, and the gain of the internal negative feedback. We asked if these varied with reward using a mixed model (Fig. 5**B–E**). Reward significantly increased feedback gain (mean fitted gains = 57.5 ± sem.3.7 s^−1^ for 0p, 62.5 ± 4.1 s^−1^ for 10p, 73.3 ± 12.3 s^−1^ for 50p; slope: *t*(20) = 2.29, p = 0.033) and reduced planning noise coefficient (5.4 ± 0.6%, 4.9 ± 0.6%, 4.6 ± 0.7%; linear model, effect of reward two-tailed *t*(20) = 2.67, p = 0.015), but did not alter premotor burst neuron noise (t(20) = 1.06, p > 0.05) or oculomotor neuron noise (t(20) = 0.83, p > 0.05). One participant had a poor model fit (squared error 4 sd. greater than mean, all other participants within 1.5 sd.); excluding this participant had no effect on the significant results (Individual fits shown in Fig. S7–S8, and the individual variance and covariance components in Fig. S10). Thus reduced variability arose for two reasons: there was less variability in the instructions entering the oculomotor system, but also the negative feedback within the saccade generator was larger, correcting internal noise in the calculation of the motor command.

In order to test whether variation of the feedback gain *g* across the reward levels is critical, a two further models were fitted to the data. In the “fixed *g*” model, *g* was constrained to be constant for each subject across the three reward levels. In the “free *g*” model, an additional parameter was added to indicate the reward slope of *g*. Reward effects for all the other three noise parameters were included in both models, so we could isolate the contribution of changes in *g*. The fitted reward effect on each parameter in the “free g” model is shown (Fig. S6A). The r-squared for the “free g” model was 0.493, and for the “fixed g” model, 0.456. This shows that, as would be expected, both models fitted the data well, but the fit was better when g was free to vary across reward levels. The squared errors for each model are shown for each subject (Fig. S6B). To determine if this improvement was sufficient to warrant including the extra parameter for reward effect, the two models were compared using BIC which penalizes the “free g” model for 1 extra parameter per subject. We used *BIC* = *n*log(*RSS*/*n*) + *k*log*n*, where RSS is the residual sum of squares for each model, *n* = 300 datapoints per subject, *k* = 7 or 8 free parameters. The ΔBIC was 263, indicating strong evidence for the model including reward effects on *g*.

## 4 Discussion

We proposed that motivation can improve performance by reducing effects of neural noise, and that noise could be attenuated by investing in negative feedback to stabilise representations, but feedback signals may themselves carry an energetic cost (Fig. 1**A–C**). The cost for attenuating noise would make precise control uneconomical unless the potential gains were high, and could thus explain why motivation improves cognitive and motor performance. First we simulated the effect of corrective negative feedback on variability in a noisy linear dynamic system. Increasing the feedback gain reduced error correlation between the middle and late states of the system (Fig. 2**D**). To test how motivation reduces inter-trial variability in the motor system, we examined saccade trajectories as a function of incentives (Fig. 1**D**). Incentives increased velocity for a given amplitude of movement, and reduced endpoint variability (Fig. 3**B,C**). Variability reduction by reward was characterised by a reduction in correlation of the saccade endpoint with variability observed in the middle parts of the movement (Fig. 2**H**), matching simulations. Fitted parameters of a model of saccade variability demonstrated that reward increased feedback gain and reduced planning noise (Fig. 5).

Reward did not reduce error correlation between the initial moments of a saccade and the rest of the saccade (Fig. 2**F**, blue zones do not extend to early timepoints). Although there is evidence for correction of early noise (i.e. the component of trial-wise variability that is present during the initial acceleration phase) during the saccade (Fig. 2**E**, blue areas at lower left and top right), this correction is not modulated by reward. In contrast, reward does increase the velocity and acceleration from the very initiation of saccades. And if anything, there was a weak *increase* in variability in the early parts of the movement with reward (Fig. 2**F**, red, significant for the 8 degree target). This increase is an expected consequence of signal-dependent noise, with faster and larger movements. So if the oculomotor system holds as a model for cognitive and motivational control in general, our results suggest that reward exerts enhancing effects on speed in the very earliest moments of processing, but then increases precision by ongoing online adjustment of task performance.

The reward-related increase in precision was significant when reward was small (0p vs 10p), but not when it was larger (10p vs 50p) (Fig. 3**C**). This diminishing effect of reward is in fact predicted from the precision-cost model (Manohar et al., 2015) when people do not value time too much (i.e. low temporal discount rate), and arises because the payoff for increasing precision saturates and is outweighed by its cost, when the requirement for accuracy is lax (Fig. S11C). Although the noise-reduction is a small effect, it is a large departure from the predictions of signal-dependent noise, where faster movements must be more variable.

### 4.1 Origin of corrective feedback signals

The online corrections observed in eye movements are too rapid to be based on visual inputs, but rather the feedback signals are thought to originate within the brain, from the generated motor signals themselves, sometimes termed “efference copy” or “corollary discharge” (Chen-Harris et al., 2008, Joiner et al., 2010). Extraocular muscles do not possess muscle spindles, so the observed effects of motivation are unlikely to be mediated by an increase in stretch reflex gain (Keller and Robinson, 1971, but see Dancause et al., 2007). One possibility is that the cerebellum may play a role in rapid error correction, particularly in adjusting the final moments of a saccade (Porrill et al., 2004). We therefore suggest that reward amplifies brainstem error-correction signals while the saccade command signal is being generated.

One alternative interpretation that cannot easily be ruled out is that reward alters the dynamics of the plant, for example by muscular co-contraction. Models of oculomotor control have generally presupposed that the eye muscles (the plant) have fixed dynamics. Increasing muscle stiffness could potentially cancel noise arising in the muscle itself, and could lead to precision improvements. However, plant stiffness would not be able to attenuate the effects of noise arising upstream from the motor command itself, which is the major source of variability in eye position (Van Gisbergen et al., 1981). Furthermore we found no evidence that reward increased co-contraction in the fixation movements that preceded the saccade (Fig. S2).

Trajectory model fits demonstrate a dual effect of reward: not only does it amplify corrective feedback, but it also improves planning, i.e. target selection, which is also susceptible to noise (Fig. 5**B,E**, Eggert et al., 2016). Fitted parameters indicate that reward acts at multiple levels, for example altering computations that determine spatial attention and arousal (Anderson et al., 2011, Hickey et al., 2010). Such higher-level cognitive factors contribute to the overall precision, and we hypothesise that this is evidence for costly noise correction mechanisms at higher cognitive levels. For example, planning precision may reflect increased stability of the ‘on-task’ state, implemented by attenuation of both internal and external cognitive noise. Resistance to such disturbances or distractors is usually termed attention, and presumably relies on similar attractor stability mechanisms (Rolls et al., 2008). But our data in this study cannot confirm this is why cognitive improvements are costly. For example, it is not clear that cognitive negative feedback signals must be encoded in a way such that larger corrections are more costly. But this would seem the most efficient coding strategy, with errors coded as additional neural activity, for similar reasons that motivate sparse or predictive coding (Barlow, 2001, Olshausen and Field, 2004).

### 4.2 Application of the theory to cognitive precision

Theories of the cost of computation hold expected reward, opportunity and energy as key economic variables that must be estimated and compared, to govern deployment of cognitive effort (Lieder et al., 2018, Shenhav et al., 2013), but not all of these factors are easily underwritten by physical costs. The present work could implicate mechanisms that stabilise neural representation, as part of these costs. We suggest that attractor stability could provide a physiological basis for the cost of precise computation. Our simplified linear feedback simulation demonstrated a system whose job is to maintain a signal constant over time. But in principle, for any neural signal transformation that can be inverted, errors in the output can be used to attenuate the effects of noise that arises during the computation (Fig. 1**C**).

We have previously suggested that the cost of internal control signals could be considered in the same manner as motor control signals, e.g. as the time integral of the squared control command (Manohar et al., 2015). Thus, a similar a cost for correcting noise can potentially be applied to any situation where cognitive precision is required, and a quantitative model can be formulated for the impact of noise. For example, noise in evidence accumulation in models of decision-making (Wang, 2002), the decay of working memory stored in attractor networks (Compte et al., 2000, Koyluoglu et al., 2017), and noise in models of inference (Fiser et al., 2010), might all be reduced by corrective signals but at a cost. Further examples might include spatial coordinate transformations, attentional selection, or higher-order planning. In these cases, it remains unclear whether cognitive signals would be subject to the same kind of analysis as motor plans (Amit, 1994). A major future challenge will be to search for such error-correction signals in more complex tasks, which can be amplified by motivation. Applying this cost function could potentially make explicit predictions about how motivation should improve precision in those domains, including parametric effects of reward and timing, and perhaps subjective effort.

It might be surprising that the low-level properties of saccades can be modulated by complex cognitive signals such as motivation by monetary reward. After all, the saccadic system is only partially cognitively penetrable: most saccades are not generated consciously. Saccades in our study were intended to be voluntary, but it is likely that even non-voluntarily-generated saccades are modulated by reward (Reppert et al., 2015). Prospects of reward may lead to improved performance even when there is no requirement to perform well (Manohar et al., 2017). Moreover, similar motivational improvements are observed in animals for both precision (Takikawa et al., 2002) and movement speed (Niv et al., 2007). These considerations all suggest that the effects may be driven by a relatively primitive system, for example ascending neurotransmitters including dopamine. Generic mechanisms such as arousal could be responsible, so that reward facilitates global improvements across many domains of function.

### 4.3 Possibility of non-neural costs

The proposed model of costly feedback control signals makes the prediction that precise internal representations require a greater overall neural activity, and would be accompanied by greater brain metabolic consumption. This seems to accord with PET and BOLD studies that demonstrate increased brain metabolism when tasks are complex, or when performance must be greater (Raichle, 1997). However, our current formulation assumes that *metabolic* costs are the primary reason that we do not always perform well. An alternate and arguably more plausible view is that the same neural mechanisms could be used for other purposes, and therefore precise cognition is limited by *opportunity* costs, rather than neural energetic costs (Kurzban et al., 2013, Westbrook and Braver, 2015). Selective attention may be a clear example of how this operates: processing one stimulus while ignoring another is hazardous. Thus, if there are processing bottlenecks where certain neurons have a “choice” over what information to encode, the resulting capacity limit would suffice to explain the ecological cost. However outside of visual cortex, it remains to be demonstrated how single neurons might be repurposed dynamically to represent different things according to needs. If it can be demonstrated that stabilising an attractor carries opportunity costs, (e.g. if fewer alternative things can be done, or perhaps behaviour is ecologically less flexible (Fallon et al., 2016)) then our predictions would still hold, albeit for a different reason.

In conclusion, we propose that motivation can improve performance by mitigating effects of noise via increasing the gain of internal feedback. This has the effect of stabilising internal representations to keep them closer to their attractor fixed-points, in the face of noise. We present preliminary evidence that motivation increases this corrective gain in the oculomotor system, indicating a cost for controlling noise.

## Funding

MRC MR/P00878X to Sanjay G Manohar, Wellcome Principal Fellowship (WT 098282) to Masud Husan, Wellcome Clinical Research Training Fellowship (WT 104364/Z/14/Z) to Kinan Muhammed.
