# Supplementary material for: Motivation dynamically increases noise resistance by internal feedback during movement
Source: Neuropsychologia. 2019 Feb 4;123:19–29. doi: 10.1016/j.neuropsychologia.2018.07.011 (PMC6363982; doi:10.1016/j.neuropsychologia.2018.07.011)
Supplement: Supplementary file 1 — Supplementary material [file mmc1.pdf]

Supplementary material

Simulating reduction of noise by feedback

Optimal control theory is a framework often applied in motor control, that allows us to determine the motor commands that need to be generated to satisfy certain constraints, or minimise certain costs. Constraints and costs may include keeping the body close to a desired trajectory in the face of noise, while also minimising the size of the motor command signals – which are treated as energetically expensive. The framework of optimal control is sufficiently general that it can be applied to abstract system states.

Let the state of a system be represented by a vector **x** at time *t*. The subsequent state is computed from the previous state, and depends on three components. First, there is a natural time evolution of the system, denoted by operator **A**. Second, the system can be kept close to its set point by applying a control signal **u**. Third, the state is corrupted by noise ε:

$$\mathbf{x}(t) = \mathbf{A}\mathbf{x}(t\text{-}1) + \mathbf{B}\mathbf{u}(t) + \mathbf{C}\varepsilon \qquad (1)$$

The magnitude and structure of the noise is determined by a matrix **C**, where ε represents an independent Gaussian random variable. **B** is an operator that determines the effect of control signals; the size of the control signal **u** must be optimised depending on the need for precise control.

How can errors be detected and corrected? In *motor* control systems, the deviations in the current physical state of the body are estimated from sensory input, which is in turn a noisy transformation of the true physical state. The sensory information can be employed to infer the true physical state, and thus to estimate the optimal motor commands **u** that would generate appropriate forces to correct the physical state (Todorov, 2005). The control signal **u** together with internal dynamics **A**, produces an attractor in which **x** sticks close to an optimal trajectory in the face of perturbations.

To find the optimum control signal **u**, a cost function can be constructed that penalises deviations of the system state from a desired trajectory (e.g. $\mathbf{x}^2$ penalises deviations from zero) but at the same time, requires the control cost $\mathbf{u}^2$ to be minimised. If the reward associated with a certain degree of error is given by the function *R*, we can write:

$$EV(\mathbf{u}) = \frac{R(\mathbf{x}^2)}{1+T(\mathbf{u})} - k\mathbf{u}^2 \qquad (2)$$

The time taken for a given motor command is given by *T*, and the energetic cost for control signals is given by *k*. Together with this cost function, optimal feedback control specifies a value of **u** that best balances the reward and the cost of being precise. Importantly, the cost of control signals is weighed against rewards, and when more rewards are on offer, this balance changes in favour of spending more on control.

A similar formalism can be applied to internal signals that may represent *cognitive* states. For example, the vector **x** might represent working memory contents, a decision variable, a goal, or an abstract state of affairs in the world. In this case, we could consider **A** to be a transformation of that state corresponding to a cognitive operation. For example, **A** might integrate evidence in a sensory channel over time to keep track of a decision variable, or it might simply hold a value constant over time. To correct for noise in these situations, it would be necessary to compare the current state with the desired state, and feed the error back into the computation.

In general, the deviation of the system from the desired state trajectory cannot be directly calculated without knowing in advance the correct result of the computation. Instead, error in the computation must be estimated from the system's new state, by a backwards computation i.e. $\mathbf{A}^{-1}(\mathbf{x}(t)–\mathbf{Bu}(t))-\mathbf{x}(t–1)$. This is equivalent to inverting the sensory transformation to obtain an updated state estimate, but now using only internal feedback (**Fig.1C**). Because saccades are *ballistic* (like many cognitive processes, internally generated without sensory feedback), they use precisely this principle to stop at the correct endpoint, despite considerable variation in earlier parts of the trajectory (Mays and Sparks, 1980; Quaia et al., 2000). Internal feedback processes such as these should therefore be able to stabilise internal representations even while a desired computation is unfolding.

We simulated a simplified linear control system to obtain qualitative predictions about trajectory variability. We used scalar **x**, and set **A**=1 indicating the system's job is to hold a value constant, in the face of noise, over 1000 timesteps. We used Gaussian noise with unit scale **C**=1, set **B**=1 so that control signals directly affect the state, and started with initial $x(1) = 0$. This corresponds to the simplest possible one-dimensional point attractor. We simulated four situations: 1) no feedback, 2) low feedback, 3) high feedback, and 4) low input noise, so that we could compare the effect of strengthening feedback gain, with the effect of simply reducing the input noise. In condition 1, we set **u**=0 indicating no corrective feedback, so that noise simply accumulates in the variable, uncorrected. To simulate this, a Gaussian random variable ε was simply added to a scalar accumulator according to the equation $dx=\varepsilon\, dt$. In condition 2, negative feedback attenuates the noise, modelled by $\mathbf{u}=-\lambda x$, giving $dx=(\varepsilon–\lambda x)dt$. We used $\lambda=0.002$. Condition 3 was identical to condition 2, but the negative feedback was strengthened by setting $\lambda=0.003$. Condition 4 was identical to condition 1, but with a lower absolute noise level, reduced by 0.2%, i.e. $dx = 0.998\ \varepsilon\ dt$.

Each scenario was simulated 1000 times. For each simulated condition we qualitatively examined the time-time autocovariance and autocorrelation. For conditions 1 and 2, **Fig 2A** and **2C** show the covariance and correlation without and with negative feedback error-correction. For condition 3, the differences (high-feedback minus low-feedback) are shown in **Fig.2B&D** right. For condition 4, the differences (low-noise minus high-noise) are shown in **Fig.2B&D** left. Similar estimates of correlation reduction with feedback gain were made from the full saccade model (**Fig.S9**).

The effect of balancing cost against reward in the simulations was demonstrated by applying a cost for the control signal of $100u^2$, and a reward dependent on error $re^{\left(-\frac{x}{100}\right)^2}$, where r is the baseline reward level. This enabled calculation of expected value using Equation (2), for various levels of feedback gain. Ignoring the cost of time, **Fig.S11A** shows the terms of equation (2), as the feedback gain λ is increased. The scale of reward r was held constant, and for a given value of r, there is an optimum feedback gain λ^* (**Fig.11B**). By increasing the reward scale r, the relative weighting of precision vs. feedback cost is tipped in favour of precision. Thus as the scale of rewards increases, the optimal feedback strength increases (**Fig.11C**), and performance improves.

No effect of reward on fixation movements before target onset

We considered whether reward might improve saccade precision by increasing co-contraction of muscles, which might manifest by changes in small ocular movements. We studied the 1400 ms period after the auditory incentive cue, before the onset of the target. To obtain clean measurements of fixation, trials which contained any saccades or blinks or any eye position that deviated by 1.8° from fixation during this period were excluded, and segments with eye velocity > 30°/s were removed. Trials were split by reward but collapsed across the three target distance conditions. On average only 101 out of 270 trials per reward level per participant (=37% ± s.d. 18%) met these stringent criteria.

First we asked whether the amplitude of ocular tremor was reduced by reward, by examining the Fourier spectrum (Bolger et al., 2000). Each trial was divided into an early period (200–700 ms after cue) and a late period (700–1200 ms), and an extended discrete Fourier transform was applied to the eye position in complex coordinates, obtaining a frequency spectrum. The average spectrum across trials for each reward level, was calculated per participant, separately for the early and late periods. These spectra are shown in **Fig S2C**. Effects of reward on the spectrum were estimated by linear regression. The log spectral power at each frequency bin was regressed against reward level. This gave estimates of the reward slope at each frequency, for each participant, which were then compared using a permuted t-statistic, corrected for multiple comparisons across frequencies. There was no significant effect of reward (all p>0.16) or interaction of reward with early/late period (all p>0.33), although numerically there was slightly lower power at 60-90 Hz in the high reward condition, in the late period. This could be consistent with a reduction in ocular tremor toward the end of the fixation period after a high-reward cue.

Second, we looked for reductions in microsaccade frequency during the same period (Josua, Tokiyama and Lisberger 2015). We examined trials with no blinks, no saccades greater than 1° in amplitude, and no eye position deviations greater than 1.8°, and examined microsaccades (amplitude less than 1°). For each participant and each reward condition, the frequency of saccades at each moment in time was estimated using the kernel-smoothed density (**Fig.S2A**). A two- to three-fold increase in microsaccade frequency was observed around 200 ms after the auditory cue. However there was no difference between the reward levels.

Third, we asked whether reward reduced the degree of ocular drift. Microsaccades were removed from the eye position traces, and the velocity was estimated in overlapping 40 ms windows. The total distance travelled per millisecond was calculated for each participant and each condition, as a function of time during the foreperiod (**Fig.S2B**). There were no effects of reward on ocular drift at any timepoint.

Supplementary References

Bolger C., Bojanic S., Sheahan N., Malone J., Hutchinson M. and Coakley D., Ocular microtremor (OMT): a new neurophysiological approach to multiple sclerosis, J. Neurol. Neurosurg. Psychiatry 68, 2000, 639–642, https://doi.org/10.1136/jnnp.68.5.639.

Todorov E., Stochastic optimal control and estimation methods adapted to the noise characteristics of the sensorimotor system, Neural Comput. 17, 2005, 1084–1108, https://doi.org/10.1162/0899766053491887

Joshua M., Tokiyama S. and Lisberger SG., Interactions between target location and reward size modulate the rate of microsaccades in monkeys, J Neurophysiol. 2015 Nov; 114(5): 2616–2624. https://doi.org/10.1152/jn.00401.2015

Summary of equations for estimating variance and endpoint-covariance trajectories for saccades, summarised from Eggert et al. (2016)

For each of the equations below, numerical integration and convolution was used to estimate quantities at the time resolution of eye position sampling (δt=0.001).

First the control signals $\bar{C}_2(t)$ and $\bar{C}_3(t)$ were estimated from the mean eye position traces $\bar{y}$ for each target distance:

$$\bar{C}_2(t) = \tau_2\tau_3 \dddot{\bar{y}}(t) + (\tau_2 + \tau_3)\ddot{\bar{y}}(t) + \dot{\bar{y}}(t)$$
$$\bar{C}_3(t) = \tau_1\bar{C}_2(t) + \int_0^t \bar{C}_2(\nu)d\nu$$

where $\bar{y}$ is the mean eye position, and τ_1, τ_2 and τ_3 are time constants of the eye-and-muscle plant system. The average amplitude of a subset of saccades is given by $\bar{A}$. Then the variance and covariance due to planning noise were estimated:

$$cov_P(t, \bar{A}) = k_A^2 \cdot \bar{A}^2 \frac{\partial y(t, \bar{A})}{\partial \bar{A}}$$
$$var_P(t, \bar{A}) = k_A^2 \cdot \bar{A}^2 \frac{\partial y(t, \bar{A})}{\partial \bar{A}}$$

where $\frac{\partial y}{\partial \bar{A}}$ was estimated from the slope of mean eye position as a function of mean saccade endpoint, estimated by linear regression. Then the variance and covariance due to premotor burst neurons was estimated using an iterative formula:

$$Q_{PBN}(t + \delta t) = \frac{k_{PBN}^2}{1 - k_{PBN}^2 \cdot m^2(0)\delta t}\left(\bar{C}_2^2(t) + \int_0^t Q_{PBN}(\nu) \cdot m^2(t - \nu)d\nu\right)$$
$$cov_{PBN}(t) = \int_0^t Q_{PBN}(\nu) \cdot q(t - \nu)q(D - \nu)d\nu$$
$$var_{PBN}(t) = \int_0^t Q_{PBN}(\nu) \cdot q^2(t - \nu)d\nu$$

And from this, the variance and covariance due to oculomotor neuron noise was estimated:

$$Q_{ON}(t) = k_{ON}^2 \cdot (\bar{C}_3^2 + \int_0^t Q_{PBN}(\nu) \cdot n^2(t-\nu) d\nu$$
$$cov_{ON} = \int_0^t Q_{ON} \cdot p(t-\nu)p(D-\nu) d\nu$$
$$var_{ON} = \int_0^t Q_{ON} \cdot p^2(t-\nu) d\nu$$

Here, the impulse response functions $p(t)$, $m(t)$, $n(t)$ and $q(t)$ depend on g, the negative feedback gain in the premotor burst integrator loop, and are given by their Laplace transforms

$$P = \frac{1}{(\tau_1 s + 1)(\tau_2 s + 1)(\tau_3 s + 1)}$$
$$M = -\frac{1}{\frac{s}{g} + 1}$$
$$N = \frac{\tau_1 s + 1}{s}(M + 1)$$
$$Q = N * P$$

except that M also includes a delay (8 msec) in the feedback loop. The impulse responses were thus computed by numerical convolution. Together, these equations gave trajectories for three variance and covariance components, which depend on four parameters: planning noise (k_A), premotor burst neuron noise (k_{PBN}), oculomotor neuron noise (k_{ON}) and the feedback gain (g). The sum of these components gives the total variance and covariance as a function of time, which are compared with the empirically observed variance and covariance. To fit the four parameters $[k_A, k_{PBN}, k_{ON}, g]$ to the eye position data, the total squared error (in degrees^4) was used as a cost function, that was minimised.

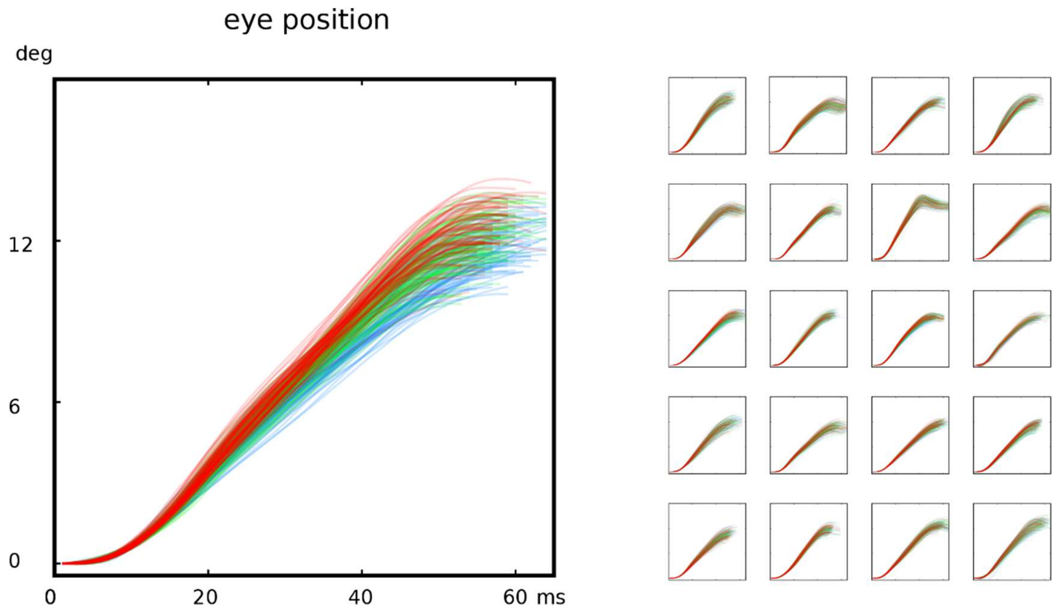

Fig S1: **Raw eye position traces for all saccades in the 12° target distance condition, for all subjects**

The raw data is shown after removing excluded trials, before normalising the movement duration. Subject number 1 is shown zoomed on the left, the remaining subjects 2 to 20 are shown on the right. Colours indicate reward level: red = 50p, green = 10p, blue = 0p (90 trials per reward level).

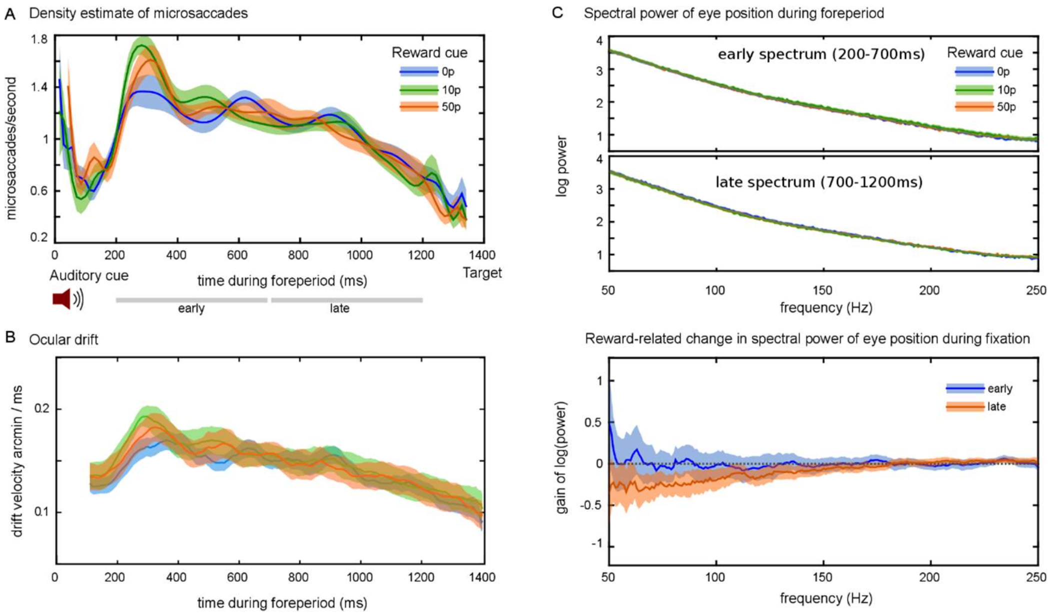

Fig. S2: No effect of motivation on fixation

These analyses examined the foreperiod after the auditory cue, and collapsed across the three target distances. A) The frequency of microsaccades was estimated at each timepoint using a kernel-smoothed density function with optimal bandwidth estimated at 210 ms. There was no significant difference in the frequency of microsaccades as a function of reward. B) Ocular drift was measured using the total distance travelled by the eye in each millisecond. This was calculated on each blink-free trial, excluding timepoints where microsaccades occurred, and the average trace was smoothed in a 100 ms window. No difference between reward conditions was found. C) Fourier spectrum of fixation movements in the early 500 ms of the foreperiod, and the late 500ms of the foreperiod. Lower panel shows the change in power at each frequency, across the reward levels (slope of linear regression). Shaded error is within-subject standard error. There were no significant effects of reward, and no significant differences in reward effect between early and late period.

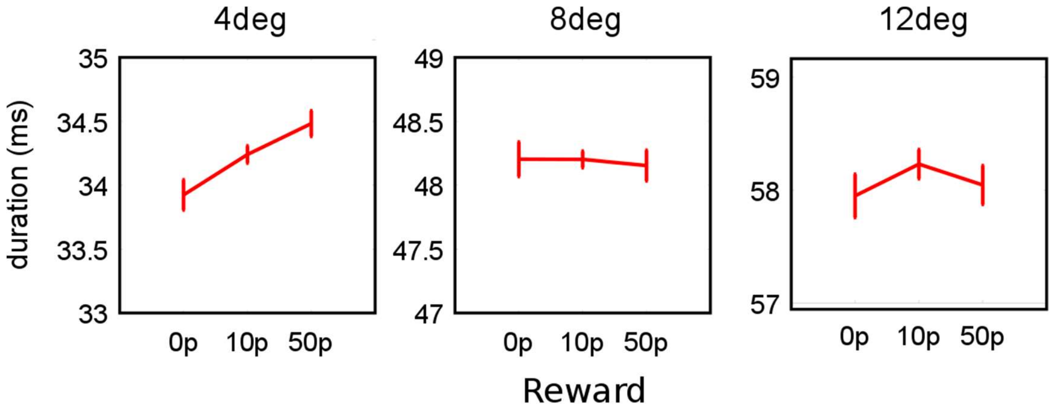

Fig. S3: No significant effect of reward on saccade duration

One possible explanation for greater correlation with higher reward, would be if saccade duration was shorter with reward, so when saccades are stretched to a comparable length, there is an artificially higher correlation between points, for high reward. Reward did consistently change saccade duration, and there was no significant interaction with amplitude.

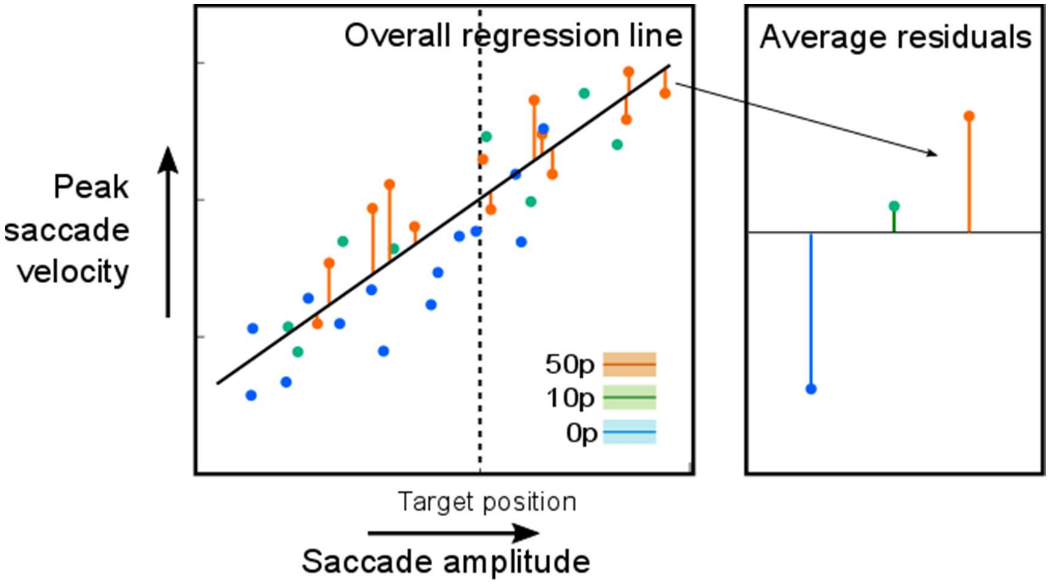

Fig.S4: Diagram of method of measuring effect of reward on the main sequence of saccades.

For a single subject and target distance condition, the saccade peak saccade velocity and corresponding amplitude is illustrated schematically (left panel). A linear regression was used to estimate the relation between peak saccade velocity and saccade amplitude, collapsed across the reward conditions (black line). The residuals of the saccade velocities (coloured line stems) were taken for each trial, indicating how much faster a particular saccade was, compared to what would be expected for its amplitude. The residuals for each reward condition were then separated out, and averaged (right panel). These averaged residuals therefore compensate for the fact the different reward conditions may have had different mean saccade amplitudes.

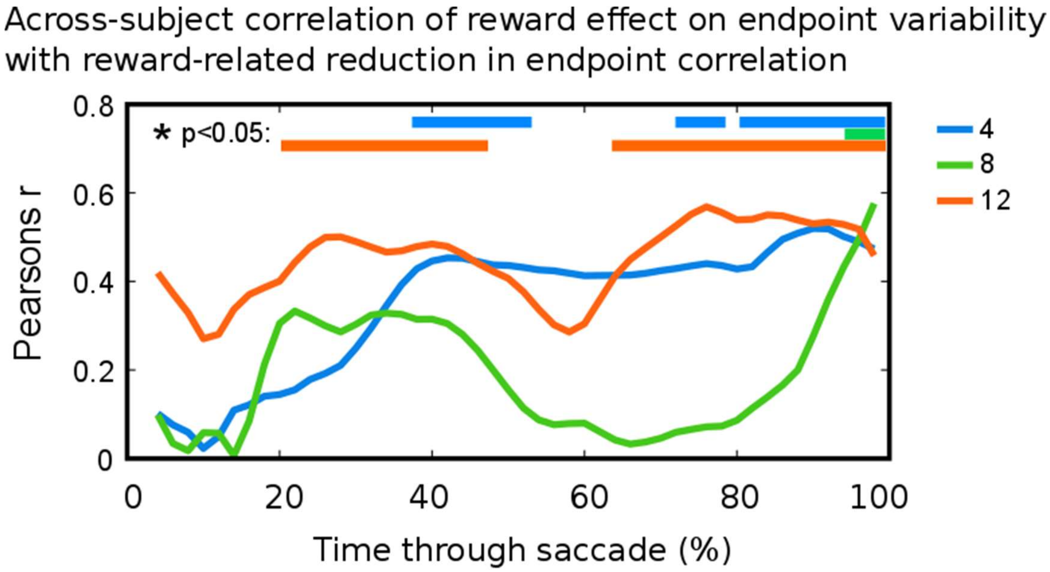

Fig. S5: Reward-related reduction of endpoint variability correlates across individuals with reward-related reduction in endpoint-correlation.

The standard deviation of endpoints (**Fig.3C**) was reduced by reward, suggesting less noise persisted to the endpoint of the saccade. We claimed that this was due to increased feedback gain during the movement as reward increased. We characterised this by reward-related reduction of the autocorrelation of eye position during the movement, particularly correlations with the endpoint of the movement (**Fig.2H**, lower or right edges of the heatmap). To strengthen this claim, we show here that across subjects, there was a relationship between these two reward effects. For each target distance, and for each timepoint during the saccade, we plot the pearson correlation between reward effect on endpoint correlation, and reward effect on the standard deviation of saccade endpoints. Bars above the plot indicate where the correlation is significant ($p<0.05$ uncorrected).

The presence of across subject correlation in these reward effects is evidence that reductions in error autocorrelation are coupled to reductions in endpoint variability. (Note that this need not be the case, even at the end of the movement, because endpoint variability could be reduced even without a change in autocorrelation.)

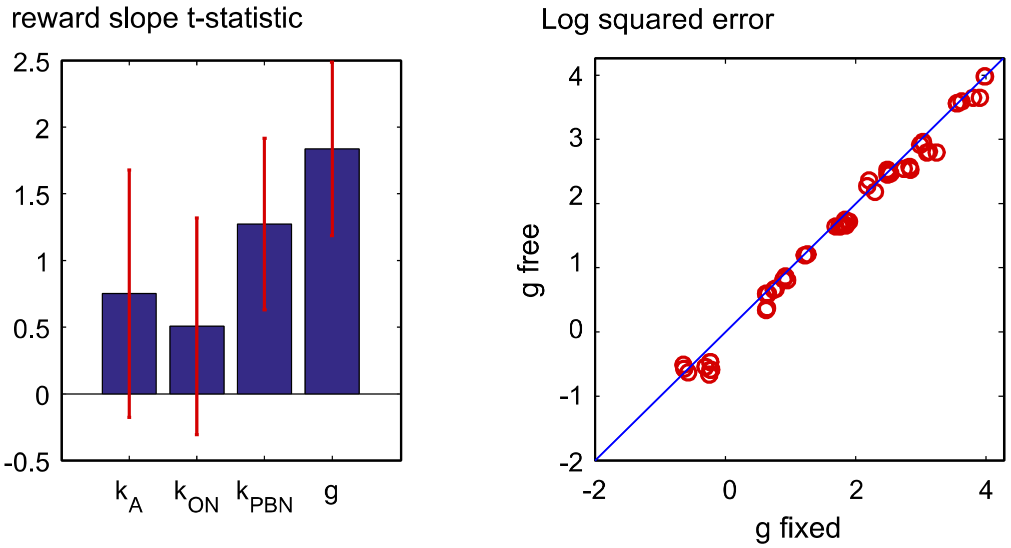

Fig.S6: Fitting models that include an effect of reward

For each subject a model was fitted that included one value for each noise parameter's base level (intercept), and a second parameter for the reward effect on this parameter (reward slope). A) The t-statistic for the reward effects was greatest for the feedback gain. B) This was compared against a model in which the gain was fixed for the three reward levels (i.e. the reward slope for *g* was constrained to be zero), but independently fitted for each subject. The squared error for each of these models was compared, and error is shown for each subject and reward level combination. As expected, the error for fixed-g tended to be larger for the error for free-g. BIC values indicated that there was strong evidence for an effect of reward on feedback gain.

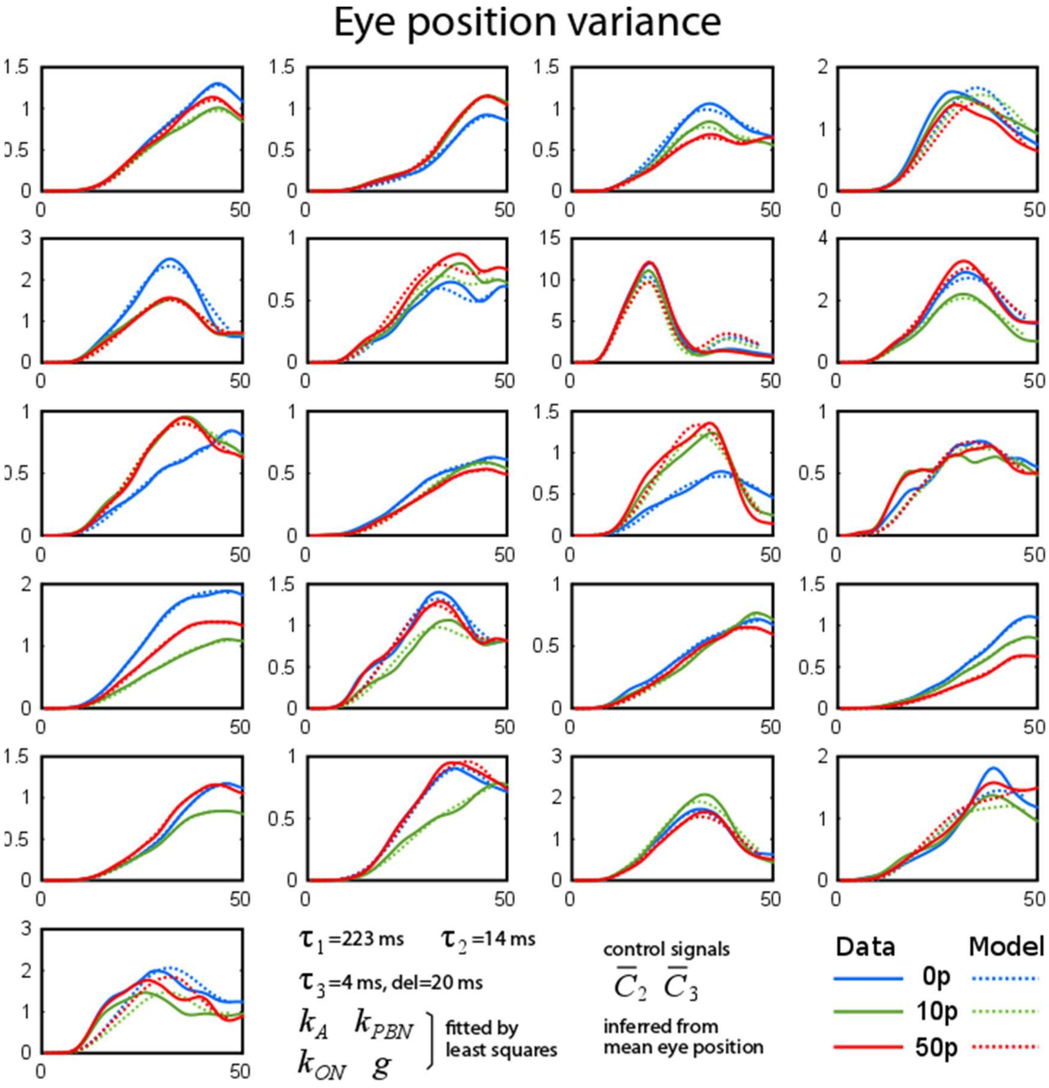

Fig.S7&8: Variance and endpoint-covariance of the eye position trajectory for each subject, compared with the model fit.

Fitting was performed simultaneously on the variance and covariance of saccades in the 12° target distance condition, using each participant's mean eye position trace as input for the variance / covariance estimate. Each reward level was fitted separately. Each panel represents one subject. Subject 7's fit (row 2 column 3) was deemed poor (total squared error more than 2 s.d. from mean), and was excluded. Note that the model predicts the variance / covariance trajectory based on the empirical mean eye position trace, which accounts for how well the modelled traces account for a number of the idiosyncratic shapes.

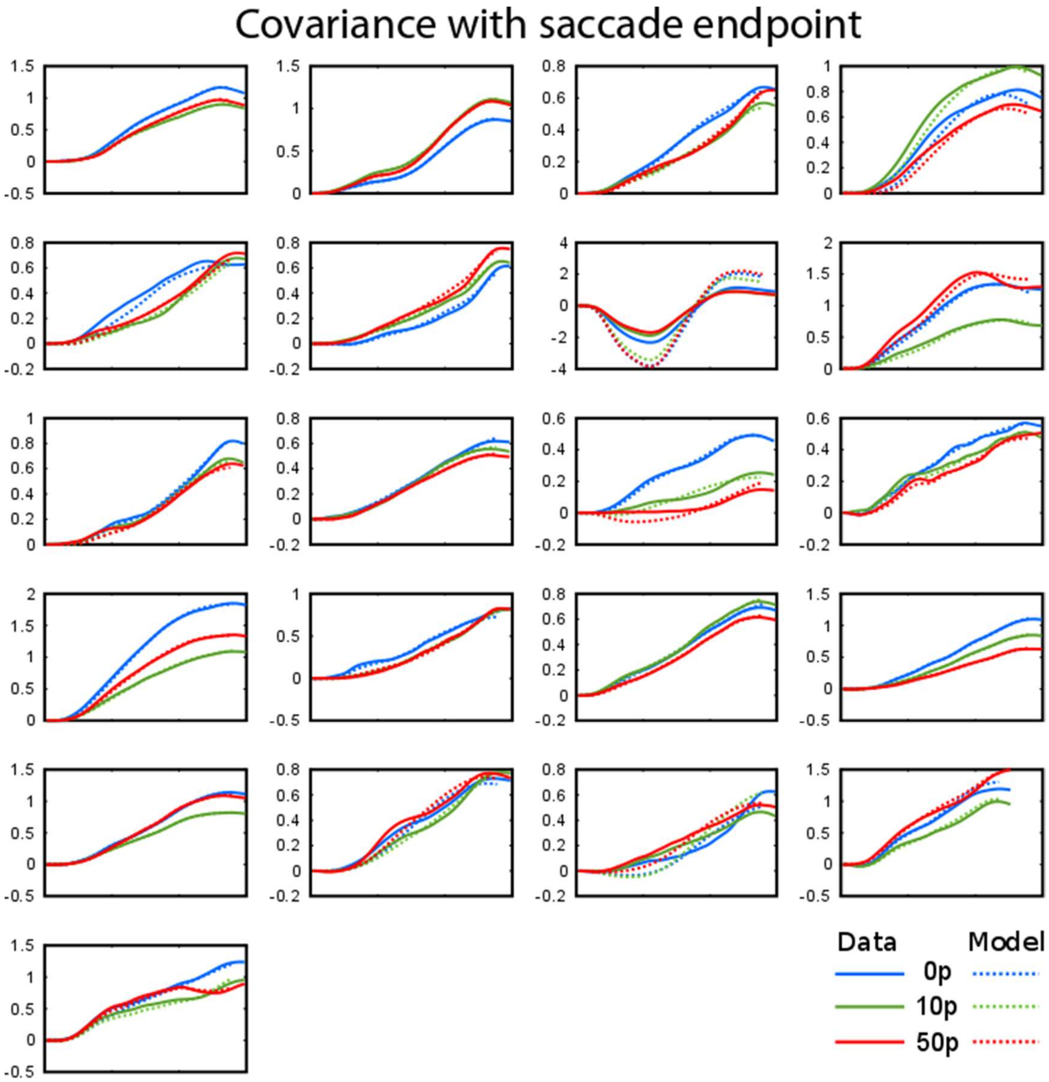

Fig. S8: see S7 for legend

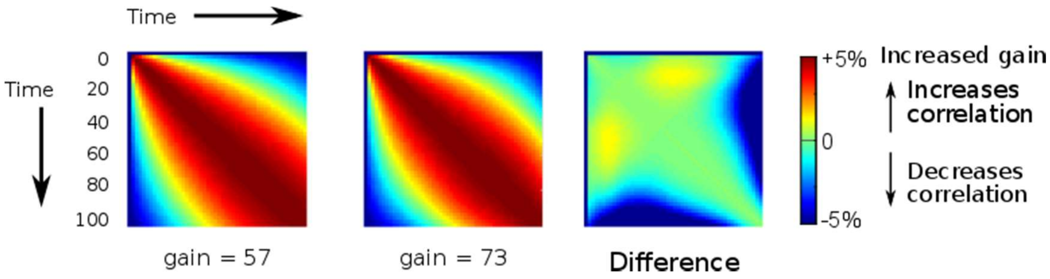

Fig.S9: Forward simulation of full saccade model with gains estimated from data

In order to demonstrate that the model of saccade dynamics recapitulates the effects on correlations that were found in the simple model, the dynamics were simulated for the empirical gain levels. Time-time correlation across trials were calculated for a numerical simulation of 10,000 trials using Euler's method. This simulation used noise levels k_A=0.001, k_{PBN}=0.010, k_{ON}=0.0001, and time parameters as in Fig.S7&8. The effect of increasing gain is qualitatively similar to the effect observed in the data.

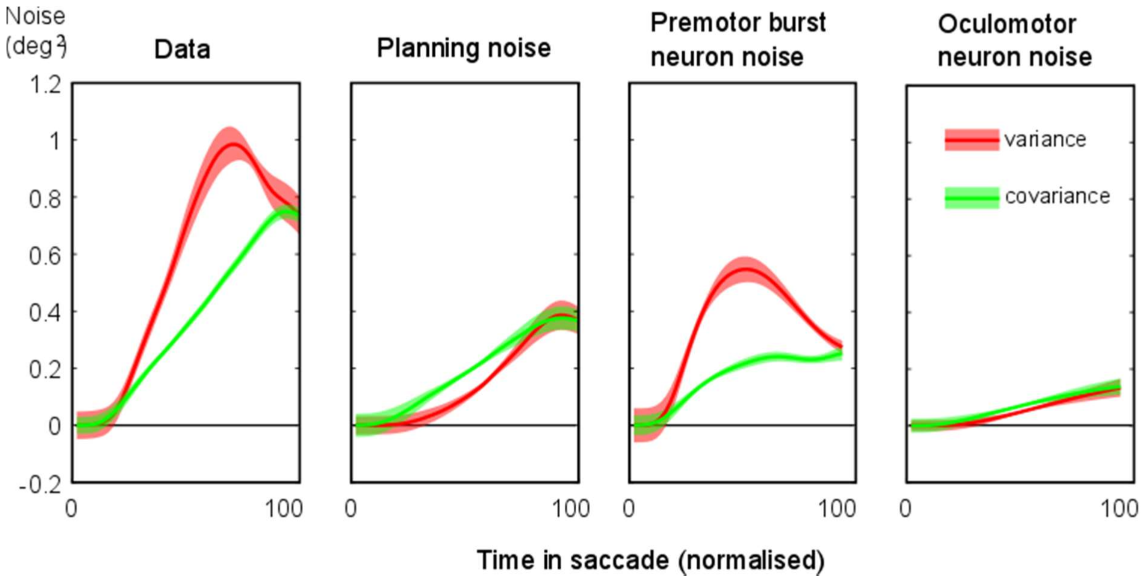

Fig.S10: Variance and covariance contributed by different modelled sources of error

The model decomposes variability in saccade trajectories into three components. Here we show each of the contributions of the noise components to the variance and covariance of trajectories. Lines are the mean across subjects, with shaded area as standard error of the mean. A) Actual variance and endpoint-covariance, measured from the eye position data. B) Modelled contribution of planning noise, C) Modelled contribution of premotor burst neuron noise, D) Modelled contribution of oculomotor neuron noise.

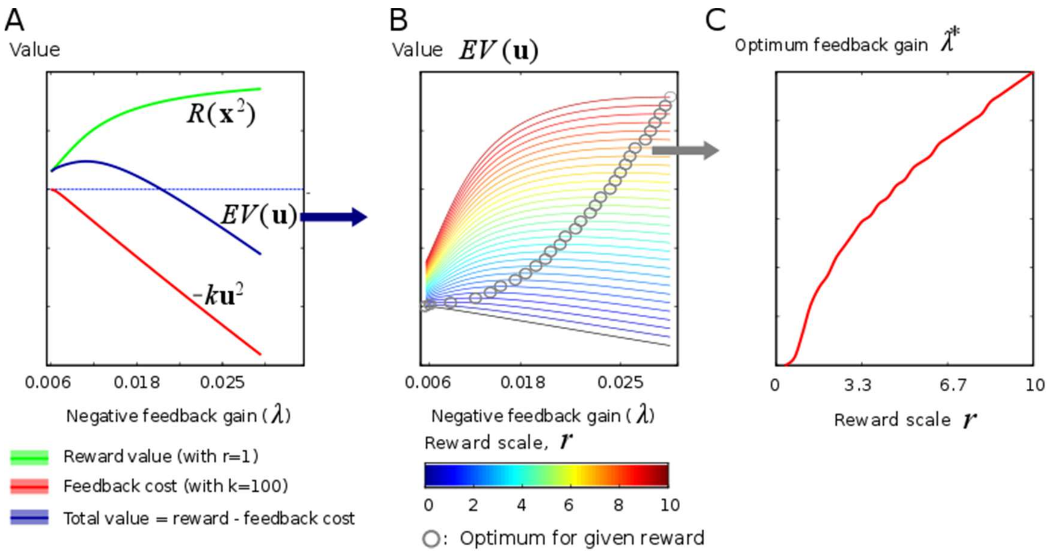

Fig.S11: Optimal values of feedback gain vary with reward

A) We examined the effect of increasing feedback gain on error, using the simplest possible linear negative feedback system (Supplementary text). Increasing feedback gain will improve accuracy (smaller x^2) but increase the feedback signal size u^2. Assuming rewards are depedent on re^{-ax^2} and cost dependent on ku^2 (taking a=k=100), there is an optimum gain that will maximise expected value. B) The value function changes shape as rewards increase, due to the relative prioritisation of reward over cost. C) This leads to the optimal gain to increase with reward. Note that as reward increases, the size of the effect of reward on optimal gain becomes shallower, a phenomenon also visible in the data (**Fig.3C**).

Size	4 deg			8 deg			12 deg		
Reward	0p	10p	50p	0p	10p	50p	0p	10p	50p
Vel ± s.e.m.	230 ± 9	235 ± 9	240 ± 9	316 ± 11	322 ± 11	326 ± 11	364 ± 12	370 ± 12	375 ± 12
Ampl	3.96 ± 0.08	4.02 ± 0.08	4.06 ± 0.08	7.92 ± 0.11	7.99 ± 0.10	8.00 ± 0.10	11.8 ± 0.15	11.8 ± 0.15	11.9 ± 0.14
Dur	33.9 ± 0.96	34.2 ± 0.93	34.5 ± 0.95	48.2 ± 1.19	48.2 ± 1.18	48.2 ± 1.20	57.9 ± 1.13	58.2 ± 1.32	58.0 ± 1.27
Var	0.62 ± 0.044	0.59 ± 0.045	0.61± 0.045	0.71 ± 0.040	0.68 ± 0.037	0.69 ± 0.036	0.84 ± 0.033	0.77 ± 0.028	0.79 ± 0.037

Table S1: Metrics of saccades for each of the nine conditions. Vel: peak saccade velocity (deg/s); s.e.m. standard error of the mean; Ampl: saccade amplitude (deg); Dur: duration of saccade (ms), Var: standard deviation of x-coordinate of saccade endpoints (deg).
